# Comparison of Three Complementary Analytical Techniques for the Evaluation of the Biosimilar Comparability of a Monoclonal Antibody and an Fc-Fusion Protein

**DOI:** 10.3389/fchem.2021.782099

**Published:** 2021-12-06

**Authors:** Alice Demelenne, Arij Ben Yahia, Delphine Lempereur, Jacques Crommen, Anne-Catherine Servais, Ines Fradi, Marianne Fillet

**Affiliations:** ^1^ Laboratory for the Analysis of Medicines, Center for Interdisciplinary Research on Medicines (CIRM), Quartier Hôpital, University of Liege, Liege, Belgium; ^2^ Laboratory of Chemical, Pharmaceutical and Pharmacological Development of Drugs, Faculty of Pharmacy, University of Monastir, Monastir, Tunisia

**Keywords:** adalimumab, etanercept, capillary gel electrophoresis, reversed-phase liquid chromatography, size-exclusion chromatography

## Abstract

In this work, a monoclonal antibody, adalimumab, and an Fc-fusion protein, etanercept, were studied and compared to one of their biosimilars. Samples submitted to stress conditions (agitation and high temperature) were used for method development. The developed methods were also applied to samples reduced by beta-mercaptoethanol to evaluate their capability to distinguish the expected species. Capillary gel electrophoresis (CGE), reversed-phase liquid chromatography (RPLC), and size-exclusion chromatography (SEC) methods coupled with UV detection were used to analyze the biopharmaceuticals. Their complementarity was investigated. For further molecular weight determination, SEC-multi angle light scattering and RPLC-quadrupole time-of-flight were occasionally used. For adalimumab, a larger amount of fragments and aggregates was observed in the biosimilar compared with the reference product. For etanercept, more related species were found in the reference product. Those three separation techniques showed good complementarity. Indeed, RPLC enabled the separation of hydrophilic and hydrophobic degradation products. CGE provided good selectivity for several adalimumab fragments, and SEC was useful for the analysis of aggregates and certain fragments that cannot be separated by the other approaches. Moreover, those formulations were submitted to mild stress conditions (30°C, 300 rpm for 4 h) that mimic shipping conditions. No additional peak was found under these conditions for the two studied biopharmaceuticals.

## 1 Introduction

Over the last decade, biopharmaceuticals have represented one of the fastest growing classes of human therapeutics. According to the International Council for Harmonization of Technical Requirements for Pharmaceuticals for human use (ICH), biopharmaceuticals are defined as molecules that are produced in various biological systems and are used to diagnose, treat, or prevent diseases. This category includes hormones, receptors, enzymes, cytokines, and monoclonal antibodies (mAbs) (ICH, 2021).

mAbs represent a major class among protein therapeutics. Indeed, around 80 therapeutic mAbs are currently approved by the European Medicines Agency (EMA) and around 100 by the Food and Drug Administration (FDA) (Antibody society, 2021). mAbs are now standard therapeutics in oncology, transplantation, and chronic inflammatory diseases (Davis et al., 2013). They are highly complex glycoproteins of approximately 150 kDa that belong to the immunoglobulin supergene family. They are composed of two identical heavy chains and two identical shorter light chains. These chains are interlinked by a variable number of disulfide bonds (Liddell, 2013).

New classes of proteins derived from mAbs are emerging, such as antibody–drug conjugates, bispecific antibodies, and Fc-fusion proteins (Davis et al., 2013). Fc-fusion proteins are molecules formed by the fusion of the crystallizable fraction (Fc) of the IgG antibody with different molecules, such as extracellular receptor domains, enzymes, or peptides, to increase their half-life and stability (Duivelshof et al., 2021). Among the biopharmaceuticals approved by the EMA or FDA, 13 are Fc-fusion proteins (Duivelshof et al., 2021).

With the expiration of the patents for those products, biosimilars have emerged on the market. Biosimilars are defined as products that are highly similar to the reference product (Gherghescu and Delgado-Charro, 2021). The first biosimilar was approved by the EMA in 2006 and by the FDA in 2015. By June 2021, 66 biosimilars had been approved by the EMA (European Medicines Agency, 2021) and 29 by the FDA (Food and Drug Administration, 2021). Among them, 38 are biosimilars of five mAbs (adalimumab, bevacizumab, infliximab, rituximab, and trastuzumab) and four are biosimilars of one Fc-fusion protein (etanercept) (European Medicines Agency, 2021; Food and Drug Administration, 2021). In the coming years, the number of biosimilars is expected to increase significantly.

To assess the biosimilar comparability between two products, comparability studies need to be performed. Those studies include the determination of physicochemical and immunochemical properties, biological activity, purity, and quantity. If differences are observed between the two products, further clinical and nonclinical evaluations are required to evaluate the impact of those changes on the final product (European Medicines Agency, 2014). Reliable analytical techniques are, thus, essential to demonstrate the biosimilar comparability.

A U.S. Pharmacopeia chapter provides the panel of analytical procedures for IgG mAb analysis. A size-exclusion chromatography (SEC) method is recommended for high-molecular-weight (MW) species assessment and a capillary gel electrophoresis (CGE) method under nonreduced and reduced conditions for fragments assessment. For the oligosaccharide analysis, capillary electrophoresis (CE) or high-performance liquid chromatography (HPLC) coupled with fluorescence detection is recommended for N-linked oligosaccharides analysis and HPLC with amperometric detection for sialic acid determination. The principles described in the chapter can be applied to other types of antibodies, such as Fc-fusion proteins (FDA, 2021).

In this work, three separation techniques [CGE, reversed-phase liquid chromatography (RPLC), and SEC] were used to analyze a monoclonal antibody (adalimumab) and an Fc-fusion protein (etanercept). CGE was used as a complementary technique to SEC for fragment analysis. Both drugs neutralize the tumor necrosis factor (TNF)-α, which is a pro-inflammatory cytokine (Goldenberg, 1999; Bang and Keating, 2004). They are used to treat diseases, such as rheumatoid arthritis and inflammatory bowel diseases (Mitoma et al., 2018).

Degraded samples using strong stress conditions and samples submitted to reducing conditions were used to optimize the separation conditions. Those methods were then applied to compare the reference products [Humira® (adalimumab) and Enbrel® (etanercept)] with their biosimilars [CinnoRA® (adalimumab) and Erelzi® (etanercept)] (see Table 1).

**TABLE 1:** Studied biologics.

Molecule	Type	Structure Davies, (2016)	Molecular weight (kDa)Goldenberg, (1999); EMEA, (2003); Lee et al. (2019)	Reference product	Biosimilar
Adalimumab	A recombinant human IgG	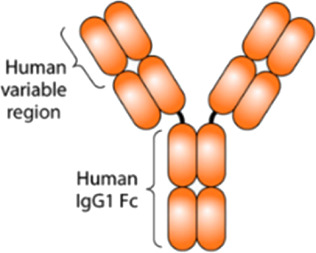	148	Humira®	CinnoRA®
Etanercept	Fc-fusion protein	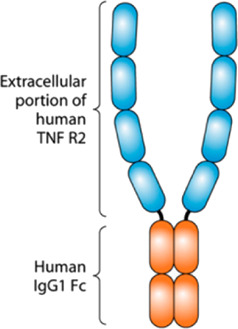	150	Enbrel®	Erelzi®

## 2 Material and Methods

### 2.1 Chemicals

Trifluoroacetic acid (TFA) ULC-MS, and formic acid (FA) ULC-MS were acquired from Biosolve (Dieuze, France). Boric acid, sodium dihydrogen phosphate (NaH_2_PO_4_), sodium dodecyl sulfate (SDS), and hydrochloric acid (HCl) were purchased from Fisher (Loughborough, Leicestershire, United Kingdom). Sodium hydroxide pellets, ethylenediaminetetraacetic acid (EDTA), disodium hydrogen phosphate (Na_2_HPO_4_), potassium chloride (KCl), and glycerol were obtained from VWR (Leuven, Belgium). Ultrapure water was obtained from a Millipore Milli-Q Academic System from Merck (Darmstadt, Germany). Ultra Gradient HPLC-grade acetonitrile (ACN) was obtained from JT Baker (Deventer, Netherlands). Tris base Ultrol Grade was purchased from Merck. Polyethylene oxide (PEO) with an MW of 200,000, phosphoric acid (H_3_PO_4_), sodium chloride (NaCl), dextran from *Leuconostoc mesenteroides* (MW 1,500,000–2,800,000), beta-mercaptoethanol (BME), lysozyme, and iodoacetamide (IAM) were acquired from Sigma-Aldrich (St. Louis, MO, United States). SDS sample buffer and SDS-MW Size Standard (10, 20, 35, 50, 100, 150, and 225 kDa proteins) were purchased from Beckman Coulter (Fullerton, CA, United States). An mAb size variant standard was obtained from Waters (Milford, MA, United States). The formulations used in this study [Enbrel® (expiration date: 12/2020), Erelzi® (expiration date: 04/2021), Humira® (expiration date: 08/2020) and CinnoRA® (expiration date: 01/2020)] were provided by a partner.

### 2.2 Instrumentation

SEC was performed on an Agilent 1200 HPLC system (Agilent Technologies, Waldbronn, Germany). RPLC was performed on an Agilent 1100 HPLC system for method evaluation (section 3.1) and on an Agilent 1200 HPLC system for biosimilar comparability exercises (section 3.2). Detection was carried out with a diode-array detector. SEC multiangle light scattering (MALS) was performed on a 1260 Bio-Inert HPLC coupled with a Bio-Dual Angle LS/DLS (Agilent). CGE-UV separations were carried out on a G7100 CE system (Agilent) coupled to a diode-array detector. For RPLC quadrupole time-of-flight (QTOF) experiments, a UHPLC system coupled to a DTIMS-QTOF mass spectrometer 6,560 (Agilent) was used. A Dual Agilent Jet Stream ESI was used as electrospray ionization source.

### 2.3 Operational Conditions

#### 2.3.1 CGE

An uncoated fused-silica capillary with a length of 33 cm (50 μm ID, 24.5 cm effective length) was used to perform all separations (Polymicro Technologies, Phoenix, AZ, United States). Hydrodynamic injection was used for all samples by applying a pressure of 4 bars for 2 s at the inlet. During the run, the pressure was set at 2 bars at both ends. The separation voltage was set at 20 kV and the UV detection wavelength was set at 220 nm. The temperature of the cassette was maintained at 25°C. Instrument control and data acquisition were achieved by using the Agilent OpenLab CDS C.01.07 (27) software.

A new capillary was first conditioned by flushing water, followed by 1 M NaOH, 0.1 M NaOH, and water again for 5 min each. The capillary was then flushed with 1 M NaOH for 5 min followed by 1 M HCl for 10 min, water for 10 min, PEO 0.2% for 5 min, and finally water for 5 min. Between each run, the capillary was rinsed with water for 3 min, 0.1 M HCl for 3 min, 0.2% PEO for 5 min, and finally with the gel for 10 min at 4 bars for each fluid. The gel was made up of 0.6 M Tris-borate buffer (pH 8.1), 0.005 M EDTA, 10% dextran (w/v), 0.2% SDS (w/v), and 10% glycerol (v/v) (Liu et al., 2008).

#### 2.3.2 RPLC

RPLC-UV: Three columns were used: BioResolve® Polyphenyl (2.1 × 100 mm, 2.7 µm particle size, 450 Å) (Waters), Biozen® intact C4 (2.1 × 50 mm, 3.6 µm particle size, 200 Å) (Phenomenex, Torrance, CA, United States), and Biozen® intact XB-C8 (2.1 × 50 mm, 3.6 µm particle size, 200 Å) (Phenomenex). The column compartment was set at 80°C for the three columns. The detection wavelength and the flow rate were, respectively, set at 280 nm and 0.4 ml/min. The injection volume was set at 10 µl. Agilent OpenLab CDS C.01.08 (210) software was used for system control and data acquisition. Mobile phase A consisted of water with 0.1% of TFA, and mobile phase B consisted of acetonitrile with 0.1% of TFA. The gradient started at 75% A and was carried out as follows: 0–20 min, from 75% to 55% A; 20–20.1 min, from 55% to 20% A.

RPLC-MS: For MS detection, the BioResolve® (polyphenyl) column was used. The same mobile phase, gradient, column temperature, and injection volume as for RPLC-UV were used. The reference masses (m/z 121.04 and 922.01) were infused at 0.1 ml/min. The Dual Agilent Jet Stream ESI source was set at a drying gas temperature and flow of 290°C and 13 L/min, respectively. The sheath gas temperature was set at 400°C and the corresponding flow at 12 L/min. The nebulizer was fixed at 20 psi. The capillary voltage was set at 5000 V and the nozzle voltage at 2000 V. The m/z range was set from 100 to 7000, and the scan rate was set at one spectrum/s. The spectra were deconvoluted using the maximum entropy algorithm in MassHunter BioConfirm software (Agilent).

#### 2.3.3 SEC

SEC-UV: BioResolve® SEC mAb column (7.8 × 300 mm, 2.5 µm particle size, 200 Å) (Waters) with a BioResolve® SEC mAb Guard column (4.6 × 30 mm, 2.5 µm particle size, 200 Å) (Waters) were used. The column compartment was thermostated at 20°C, and the wavelength was set at 280 nm. The flow rate and injection volume were set at 0.5 ml/min and 10 μl, respectively. Agilent OpenLab CDS C.01.08 (210) software was used for system control and data acquisition. The mobile phase was composed of 50 mM phosphate buffer (pH 7) and 200 mM KCl.

SEC-MALS: For MALS detection, the same column and mobile phase as for SEC-UV were used. The column compartment was thermostated at 30°C, and the LS wavelength was set at 658 nm. The flow rate was set at 0.5 ml/min. The dn/dc ratio and UV extraction coefficient were found in the literature and were set at 0.185 ml/g and 1.42 ml/(mg.cm), respectively (Gokarn et al., 2015) for mAb size variant standard, at 0.185 ml/g and 1.39 ml/(mg.cm) for adalimumab, and at 0.172 ml/g and 0.912 ml/(mg.cm) (Miranda-hernández et al., 2015) for etanercept. Agilent Bio-SEC software was used for system control and data acquisition. The injection volume was 30 µl for mAb size variant standard, 40 µl for adalimumab, and 10 µl for etanercept.

#### 2.3.4 Sample Preparation

##### 2.3.4.1 CGE

CinnoRA®, Enbrel®, and Erelzi® formulations were diluted 20 times in Tris-HCl buffer at pH 9.0 with 1% of SDS (SDS sample buffer) containing 6 mM of iodoacetamide. Humira® was diluted 40 times in the same medium. All samples (at a final concentration of 2.5 mg/ml) were then heated at 70°C for 10 min. For the reduction, each formulation was diluted 50 times in a solution of 5% BME in SDS sample buffer and then heated for 15 min at 70°C.

##### 2.3.4.2 RPLC

RPLC-UV: CinnoRA®, Enbrel®, and Erelzi® were diluted 100 times in 0.1% FA to obtain a concentration of 0.5 mg/ml. Humira® was diluted 200 times in the same solvent to reach the same concentration. For the reduction, 2 µl of CinnoRA®/Enbrel®, 10 µl of BME, and 188 µl of water were mixed. The samples were then heated for 15 min at 70°C.

RPLC-MS: CinnoRA® and Humira® were diluted 100 and 200 times in 0.1% FA, respectively, and 500 µl of samples were centrifuged in Vivaspin 500® Centrifugal Concentrator of 10 kDa (Sartorius, Goettingen, Germany) during 5 min at 12,000 rpm. Then, 20 µl of the nonfiltered liquid was collected, and 180 µl of water containing 1% FA was added.

##### 2.3.4.3 SEC

SEC-UV: CinnoRA®, Enbrel®, and Erelzi® were diluted 100 times in water, and Humira® was diluted 200 times to achieve a final concentration of 0.5 mg/ml. For the reduction, 2 µl of formulation were diluted 100 times in a 10% BME solution and then heated for 15 min at 70°C.

SEC-MALS: CinnoRA® and Enbrel® were diluted 25 times in water to reach a concentration of 2 mg/ml.

##### 2.3.4.4 Forced Degradation

For this, 350 µl of CinnoRA® and 200 µl of Enbrel® were subjected to stirring (600 rpm) at 60°C for 2 h. These samples were then prepared as mentioned above.

#### 2.3.5 Calibration

##### 2.3.5.1 CGE

Standard of MW preparation: 5 µl of the SDS-MW size standard (10, 20, 35, 50, 100, 150, and 225 kDa proteins) were mixed with 5 µl of BME and 70 µl of SDS-sample buffer. The standard was heated at 100°C for 3 min.

The waters mAb size variant standard preparation: The waters mAb size variant standard was diluted two times in SDS-sample buffer before injection.

CGE calibration curve: The curve was made using proteins from the standard of MW at 20 and 35 kDa and the main peak at 148 kDa from the waters mAb size variant standard (see Supplementary Figure S1).

##### 2.3.5.2 SEC

The Waters mAb size variant standard was composed of main peak (148 kDa) low-MW species around 50 and 100 kDa, and high-MW species larger than 300 kDa were used for MW estimation.

### 2.4 Statistical Analysis

Statistical analyses were performed using GraphPad Prism 6 (GraphPad Software, Inc. La Jolla, CA). Two-way ANOVA tests were performed.

## 3 Results and Discussion

### 3.1 Method Evaluation Using Samples Submitted to Stress and Reducing Conditions

Adalimumab and etanercept were submitted to forced degradation conditions. Usually, forced degradation studies are performed to determine the degradation pathways of biopharmaceuticals and their stability (Venkataraman and Manasa, 2018). Here, we used degraded samples to assess the selectivity of the analytical methods (ICH, 1996). Each formulation was submitted to high temperature (60°C) and agitation (600 rpm) for 2 h. This process was expected to denature the proteins and generate aggregates, fragments, oxidation, and deamidation products (Tamizi and Jouyban, 2016; Nowak et al., 2017; Le Basle et al., 2020).

Moreover, BME, a reducing agent, was added to adalimumab and etanercept samples to cleave the disulfide bridges of the proteins (Müller and Winter, 2017). Intact adalimumab MW is approximately 148 kDa (EMEA, 2003; Lee et al., 2019). The treatment with BME was supposed to give two peaks for adalimumab corresponding to the light (25 kDa) and heavy (50 kDa) chains. Intact etanercept MW is approximately 150 kDa (Goldenberg, 1999). For etanercept, two fragments of about 75 kDa should be generated (see Supplementary Figure S2).

For method optimization, adalimumab biosimilar and etanercept reference product formulations were used. They were analyzed before and after stress conditions (temperature and agitation) and reduction by CGE, RPLC, and SEC.

#### 3.1.1 RPLC

Three core-shell columns with a solid core and a porous outer layer were tested by RPLC. The stationary phases of the Biozen® columns (C4 and XB-C8; 3.6 µm particles) are grafted with alkyl groups and endcapped with trimethylsilyl groups. This reduces the interaction between the positive charges of adalimumab and etanercept and free silanols that may be negatively charged at a pH above 3. The BioResolve® column is functionalized with polyphenyl groups. This type of grafting has recently been used for the separation of antibodies and derived products (Bobály et al., 2018). The particles have a diameter of 2.7 µm. Moreover, the BioResolve® column used in this study is two times longer than the Biozen® columns.

Gradient slope and flow rates were optimized (data not shown). Not surprisingly, the BioResolve® column offered the best performance. As shown in Supplementary Figure S3, the column was thermostated at 80°C as it is well known that a high temperature decreases the phenomenon of adsorption of monoclonal antibodies on the stationary phase (François and Guillarme, 2020). Working at 60°C was not high enough because an additional peak was still observed (Supplementary Figure S3A). At 80°C, this additional peak almost disappeared. This phenomenon was much less pronounced for etanercept samples (Supplementary Figure S3B).

**FIGURE 1:**
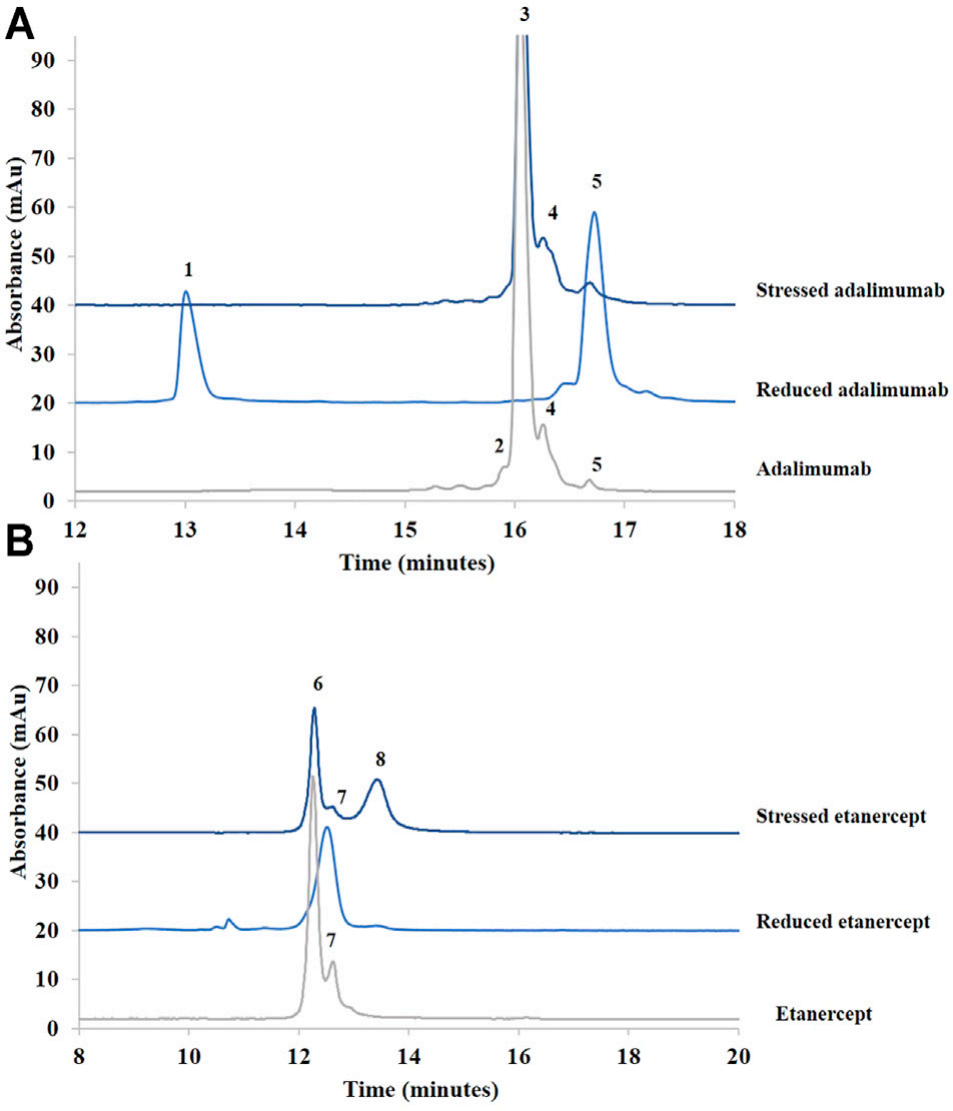
Chromatograms of treated (stressed and reduced) and untreated samples of adalimumab **(A)** and etanercept **(B)** obtained by RPLC. See Material and methods for additional information on chromatographic conditions. 1. Light chain; 2. More hydrophilic species; 3. Adalimumab; 4. More hydrophobic species; 5. Heavy chain; 6. Etanercept; 7.75 kDa fragment; 8. More hydrophobic species.

Figure 1 shows the chromatograms obtained for adalimumab and etanercept under reducing and stress conditions. Under stress conditions, the small peaks already present in adalimumab (peaks 4 and 5) were still present, but the shoulder before the main peak (peak 2) corresponding to hydrophilic species disappeared.

After reduction, the chromatogram of adalimumab shows that the light (peak 1) and heavy chains (peak 5) are well separated (Figure 1A). The light chain, which is more hydrophilic, elutes first. The heavy chain elutes after the intact antibody (Bobály et al., 2018). This suggests that hydrophobic moieties in the heavy chain are hidden when the light chain is bound. Once the two chains are separated, these hydrophobic moieties could be exposed, which might explain the higher retention of the heavy chain compared with the intact antibody.

Etanercept was analyzed under stress conditions as well. A major peak, more retained than the main peak of untreated etanercept, was generated (peak 8). For this recombinant protein, BME cleaves the disulfide bridges at the hinge region. The molecule should be divided into two identical fragments of about 75 kDa (see Supplementary Figure S2). Because there are intrachannel disulfide bridges, the three-dimensional conformation of the fragments is also modified. As can be seen in Figure 1B, the generated peak is slightly more retained than the major peak observed in the nondegraded sample.

In sum, RPLC gave information on the hydrophobic character of recombinant proteins and their degradation products. Moreover, this technique was able to separate the species obtained after sample reduction. Further investigations concerning the identity of these species are discussed in section 3.2 dedicated to the biosimilar comparability study.

#### 3.1.2 CGE

**FIGURE 2:**
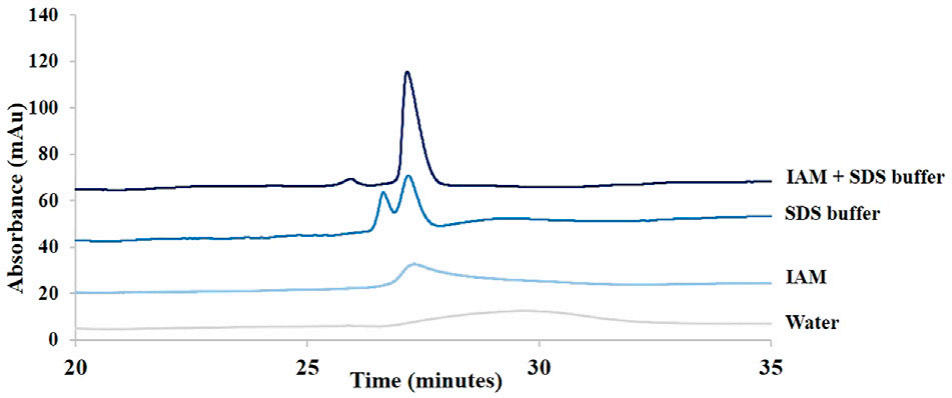
Influence of the dilution solvent on adalimumab peak shape in SDS-CGE. Comparison of samples diluted 20 times in water, in 5 mM IAM, in SDS buffer, and in a mixture of SDS buffer and IAM. See Material and methods for additional information about CGE conditions.

An SDS-CGE method previously developed for the study of protein aggregation was used (Demelenne et al., 2019). The nature of the sample dilution solvent was optimized using adalimumab. This solvent appeared to have a tremendous impact on antibody peak shape (see Figure 2). Samples were first diluted in a Tris-HCl buffer at pH 9.0 with 1% of SDS and then heated. SDS is a denaturing agent that binds to the hydrophobic residues of the protein and disrupts noncovalent bonds and secondary and tertiary structures (Duivelshof et al., 2021). Covalent bonds, such as disulfide bridges or peptide bonds, remain intact (Schmid et al., 2017). SDS is used to give uniform charge to the molecules, allowing for a size-based separation in SDS-CGE.

Figure 2 shows the impact of the composition of the dilution solvent on adalimumab peak shape. As can be seen in this figure, it was necessary to block the free thiol groups of cysteine to analyze intact antibodies. Indeed, free thiol groups can reduce neighboring disulfide bonds, which can lead to the formation of unwanted species (Zhang et al., 2010). IAM is an alkylation agent that binds specifically to free thiol groups and blocks them. IAM prevents, thus, the formation of intermolecular disulfide bridges and the cleavage of existing disulfide bonds (Salas-Solano et al., 2006). Without IAM in the sample buffer, two peaks were detected, and with IAM in the buffer, one main peak was detected.

**FIGURE 3:**
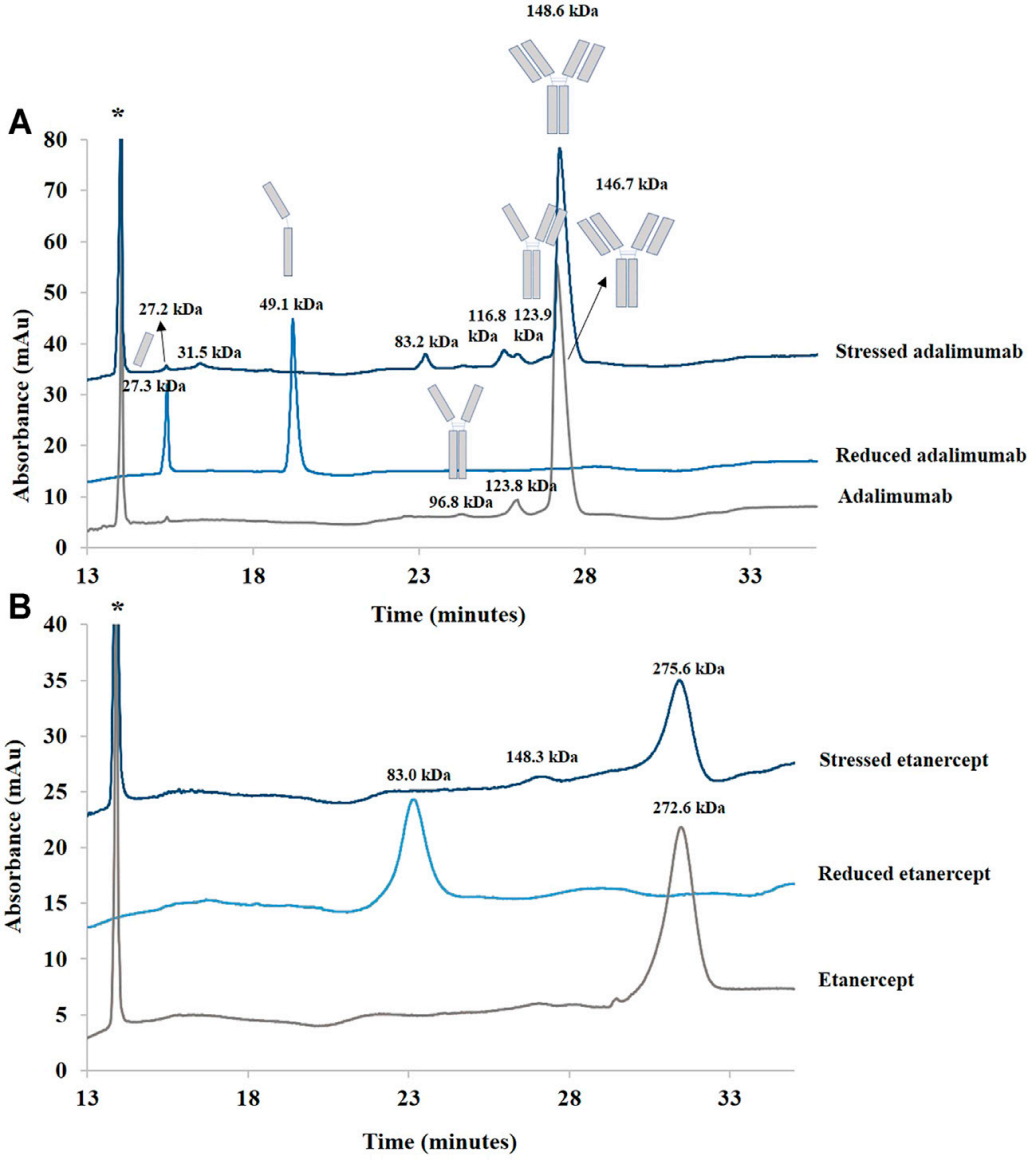
Electropherograms of treated (stressed and reduced) and untreated samples of adalimumab **(A)** and etanercept **(B)** obtained by SDS-CGE. Molecular weights were estimated using a calibration curve. See Material and methods for additional information on the CGE conditions. *: lysozyme (internal standard).

Adalimumab and etanercept were, thus, analyzed under those conditions. Lysozyme was used as an internal standard to correct peak areas and migration times. Adalimumab had a migration time of 27.2 min, and etanercept migrated at 31.5 min (see Figure 3). The MWs of the studied biopharmaceuticals were estimated using a calibration curve (see Material and methods). MWs of adalimumab and etanercept were estimated at 146.7 and 272.6 kDa, respectively. This is pretty close to the actual MW for adalimumab (148 kDa) but not for etanercept (150 kDa). This could be explained by the extended conformation of the sugar on etanercept. As etanercept is highly glycosylated, the protein could be not fully recovered by SDS, and the migration time is, thus, longer than for a molecule of similar MW with less glycosylation. This results in a biased MW determination (Wang et al., 2017). Moreover, etanercept appears as a broad peak due to the heterogeneity of the glycans that represent one third of its total MW.

For the samples treated with BME, two peaks (at 15.5 and 19.5 min) were observed for adalimumab as expected. MWs for the light and heavy chains of adalimumab were estimated at 27.3 and 49.1 kDa, respectively. For etanercept, the treatment of the sample with BME provided only one peak estimated at 83.0 kDa.

As can also be seen in Figure 3, the sample of adalimumab submitted to stress generated species between 25 and 150 kDa (27.2, 31.5, 83.2, 116.8, and 123.9 kDa). The 27.2 kDa corresponds to the light chain of the antibody. The 123.9 kDa species could be related to the antibody without one of its light chains (theoretical value of 125 kDa).

In the untreated sample, the light chain of the antibody was found as well. Moreover, the 96.8 kDa species could correspond to the antibody with two heavy chains and no light chains (theoretical value of 100 kDa).

For etanercept, a peak estimated at 148.3 kDa was observed after agitation at 60°C for 2 h. Moreover, there is a loss of almost 50% of the normalized peak area (ratio of the peak area to its migration time corrected with the ratio of the peak area of the internal standard to its migration time). This low recovery suggests some precipitation of etanercept before injection (but a low recovery would also be observed with other techniques in that case), some adsorption of the high-MW species to the capillary wall, or some impossibilities for the high-MW species to enter into the gel.

No peak of higher MW than adalimumab or etanercept was found. Indeed SDS-CGE is not a suitable technique for the detection of noncovalent aggregates because they would be dissociated by SDS present in the buffer and in the sample (Duivelshof et al., 2021). However, CGE was able to distinguish between species of various sizes in adalimumab and etanercept.

#### 3.1.3 SEC

Adalimumab and etanercept were analyzed by SEC coupled to UV and MALS using a previously described method (Waters, 2020). The SEC analysis was performed on a BioResolve® SEC mAb column of 2.5 µm particle size and 200 Å pore size of 30 cm with 7.8 mm internal diameter. The mobile phase was composed of 50 mM phosphate buffer (pH 7) and 200 mM KCl (Waters, 2020). An mAb standard was used for MW estimation by SEC-UV (see Section 2.3.5.2).

**FIGURE 4:**
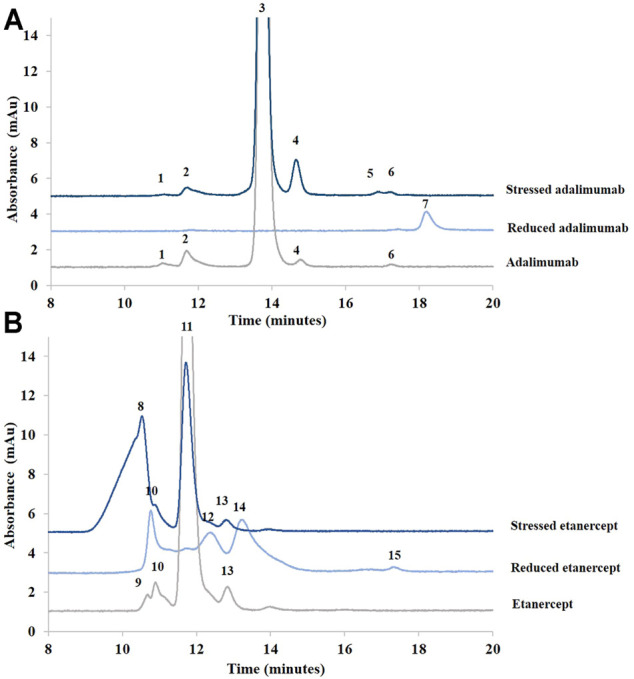
Chromatograms of treated (stressed and reduced) and untreated samples of adalimumab **(A)** and etanercept **(B)** obtained by SEC. See Material and methods for additional information on chromatographic conditions. 1, 2. Higher MW species of adalimumab (≥300 kDa); 3. Adalimumab (∼150 kDa); 4. Lower MW species of adalimumab (∼100 kDa); 5, 6. Lower MW species of adalimumab (∼50 kDa); 7. Adalimumab fragment; 8, 9. Higher MW species of etanercept; 10. Etanercept; 11–15. Lower MW species of etanercept.

As can be seen in Figure 4, a difference in retention time between adalimumab and etanercept was observed. Adalimumab eluted in 13.7 min, and etanercept eluted after only 11.7 min. This suggests that etanercept is a larger protein than adalimumab although they have almost the same molecular weight (∼150 kDa). Using a calibration curve obtained from mAb standard species, etanercept MW was estimated at 300 kDa. This is consistent with the value observed in CGE in which etanercept MW was estimated at 260 kDa, much larger than expected. It is described in the literature that sugars may have an important effect on elution in SEC and, thus, that SEC-UV may not be suitable for MW determination of highly glycosylated proteins (Wen et al., 1996).

The analysis of the species obtained after BME reduction showed one single peak at 50 kDa, which corresponds to the heavy chain. The light chain could not be detected or coeluted with the heavy chain. For reduced etanercept, four peaks were observed instead of the expected single peak.

MALS was then used as an absolute approach to assess the MW of untreated adalimumab and etanercept and after stress conditions. Adalimumab (peak 3, corresponding to the intact product), has an estimated MW of 152 kDa. This is close to the expected MW of 148 kDa. The MW of the species related to peak 1 in the stressed solution could correspond to a complex of approximately 5 adalimumab molecules (819 kDa). Peak 2 species could correspond to a trimer (455 kDa). Peak 4 species could correspond to a mixture of antibodies that have lost at least one light chain (115 kDa). Peaks 5 and 6 species could correspond to the antibody that has lost its two light chains (109 kDa). As two peaks were observed, it should probably correspond to two populations of two heavy chain antibodies with small differences in their MW. It is interesting to note that peak 4 represents only 1% of the main peak in the untreated adalimumab sample but 6.6% of the main peak in the degraded sample. The percentage of high-MW species was almost identical in both samples (the sum of peaks 1 and 2 represents 3.3% of the main peak in the untreated sample and 3.2% of the main peak in the degraded sample).

For etanercept, the MW of peak 11 species, corresponding to intact etanercept, was estimated at 151 kDa. For etanercept submitted to stress conditions, a significant increase of the aggregated species (peak 8) was observed. Indeed, this peak represents 6.5% of the main peak (peak 11) in the reference product although it represents 166% of peak 11 in the stressed sample. The MW of this aggregate was found at 3451 kDa, which might correspond to the aggregation of 23 Fc-fusion proteins. In the untreated sample, peak 9 was estimated to correspond to a trimer (464 kDa), and peak 10 was estimated to correspond to a dimer (266 kDa). Peaks 12–15 species correspond to lower MW species.

In sum, aggregates could be nicely separated from adalimumab and etanercept fragments by SEC. For adalimumab, the light chain of the antibody could not be detected. MALS, which is an absolute approach, was necessary to estimate the MW of etanercept accurately.

### 3.2 Biosimilar Comparability Exercise

In the second part of this study, biosimilars of adalimumab and etanercept were compared with the reference products to evaluate their comparability. It should be noted that both the biosimilar and the reference product of adalimumab were outdated. However, they had expired less than a year previous to the time of analysis.

The formulations were compared in their initial state and after submission to a mild stress supposed to mimic biopharmaceuticals shipping conditions, i.e., agitation at 300 rpm for 4 h at 30°C.

#### 3.2.1 Adalimumab

**FIGURE 5:**
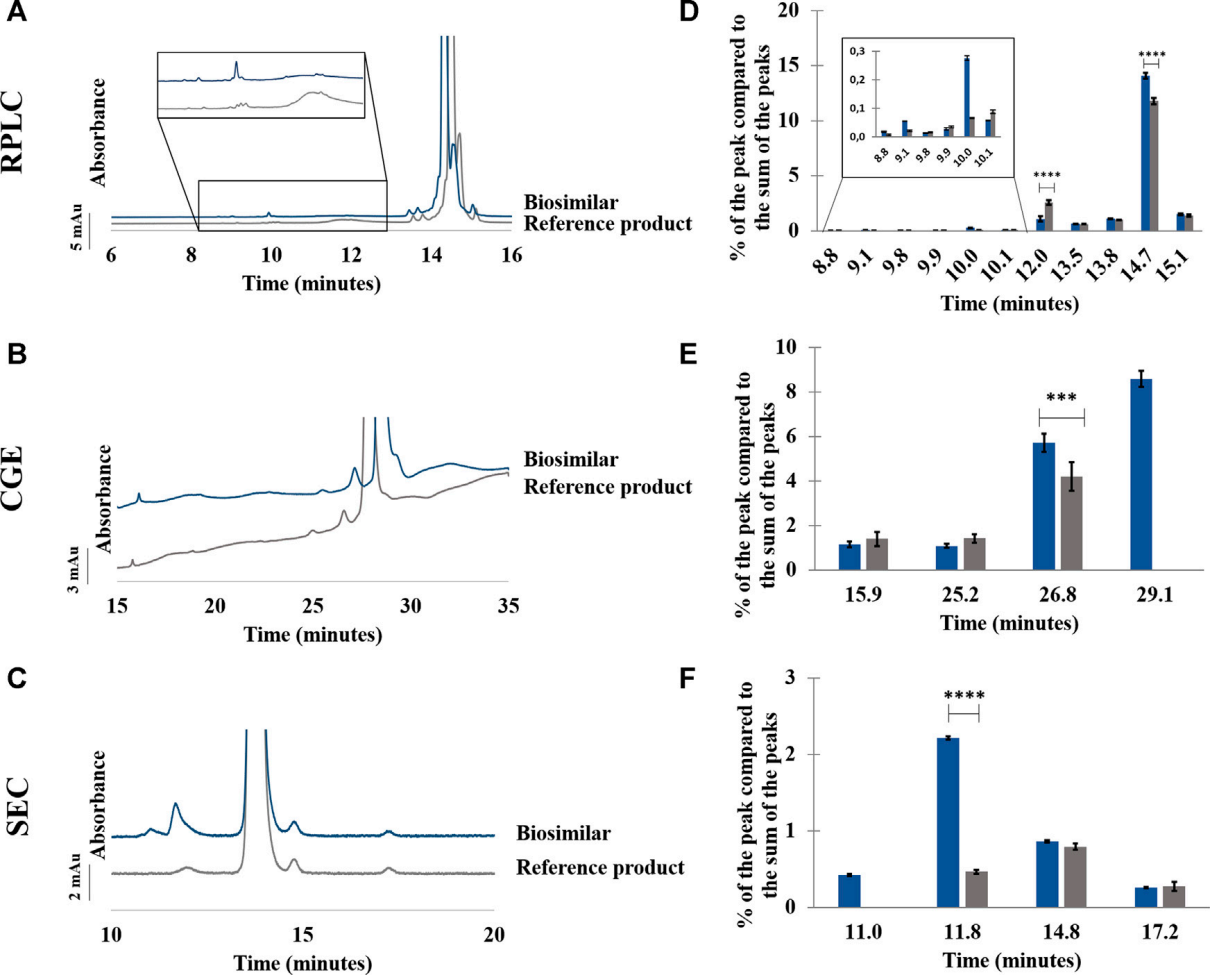
Comparison between adalimumab reference product and its biosimilar by RPLC **(A–D)**, CGE **(B–E),** and SEC **(C–F)**. Chromatograms **(A–C)** and percentage of the peak compared to the sum of the peaks in function of the retention/migration time (n = 3) **(D–F)**. For CGE, the peak areas of the peaks were corrected by their migration times. *** represents statistically different results with a *p*-value <0.001. **** represents statistically different results with a *p*-value <0.0001. See Material and methods for additional information on experimental conditions.

As can be seen in Figure 5A,D, few differences were observed in RPLC between the biosimilar and its reference product for the formulations in their initial state. The sum of the more hydrophilic impurities represents 3.3% of the sum of the peak areas for the biosimilar, and it represents 4.5% of the sum of the peak areas for the reference product. In particular, the peak at 10.1 min was more abundant in the biosimilar compared with the reference product, and the peak at 12.0 min was more abundant in the reference product than in the biosimilar (see Supplementary Table S1). Concerning the more hydrophobic impurities, they were slightly more abundant in the biosimilar as they represented 15.6% of the sum of the peak areas compared with 13.1% for the reference product. The repeatability of the method was evaluated from three injections of each product. RSDs below 0.3% were found for the retention time and peak area of the main peak for both the biosimilar and the reference product (see Supplementary Table S2).

Samples were also analyzed by RPLC-QTOF to determine the exact mass of the main peak of the reference product and that of the biosimilar. Antibodies are often characterized by their N-glycan composition. G0F glycoform is one of the most frequent types and represents oligosaccharides that contain fucose with no terminal galactosylation (Tebbey et al., 2015). Moreover, adalimumab is present as three major isoforms depending on the number of C-terminal lysine (0, 1, or 2) carried by the molecule (EMEA, 2003). The main peak for the reference product of adalimumab was detected at 148,092.25 Da and could be attributed to adalimumab G0F/G0F glycoform with no terminal lysine on the C terminal chain (Füssl et al., 2019). The main peak for the biosimilar of adalimumab was detected at almost the same MW (148,093.20 Da).

In CGE, a shoulder can be seen after the main peak in the biosimilar but not in the reference product. This shoulder represents around 8.6% of the sum of the peak areas. Moreover, the peak corresponding to the 125 kDa fragment was more abundant in the biosimilar than in the reference product (see Figure 5B,E; Supplementary Table S1). RSDs below 0.5% were found for migration times of the main peak. For peak area, 9.1% and 4.3% of variation were observed, respectively, for the reference product and the biosimilar (See Supplementary Table S2).

In SEC, a higher proportion of aggregates was found in the biosimilar than in the reference product (2.6% vs. 0.5%). Moreover, an aggregate was found at 11.0 min in the biosimilar that was not present in the reference product. Concerning the fragments, there were no significant differences between the two biopharmaceuticals (see Figure 5C,F; Supplementary Table S1). RSDs on retention time and peak area of the main peak were below 0.1% for the reference product and the biosimilar (See Supplementary Table S2).

For samples of the reference product and biosimilar subjected to a temperature of 30°C and a stirring speed of 300 rpm for 4 h, no change in the chromatographic and electrophoretic profiles was observed (data not shown). This indicates a similar stability of the product under those conditions.

#### 3.2.2 Etanercept

**FIGURE 6:**
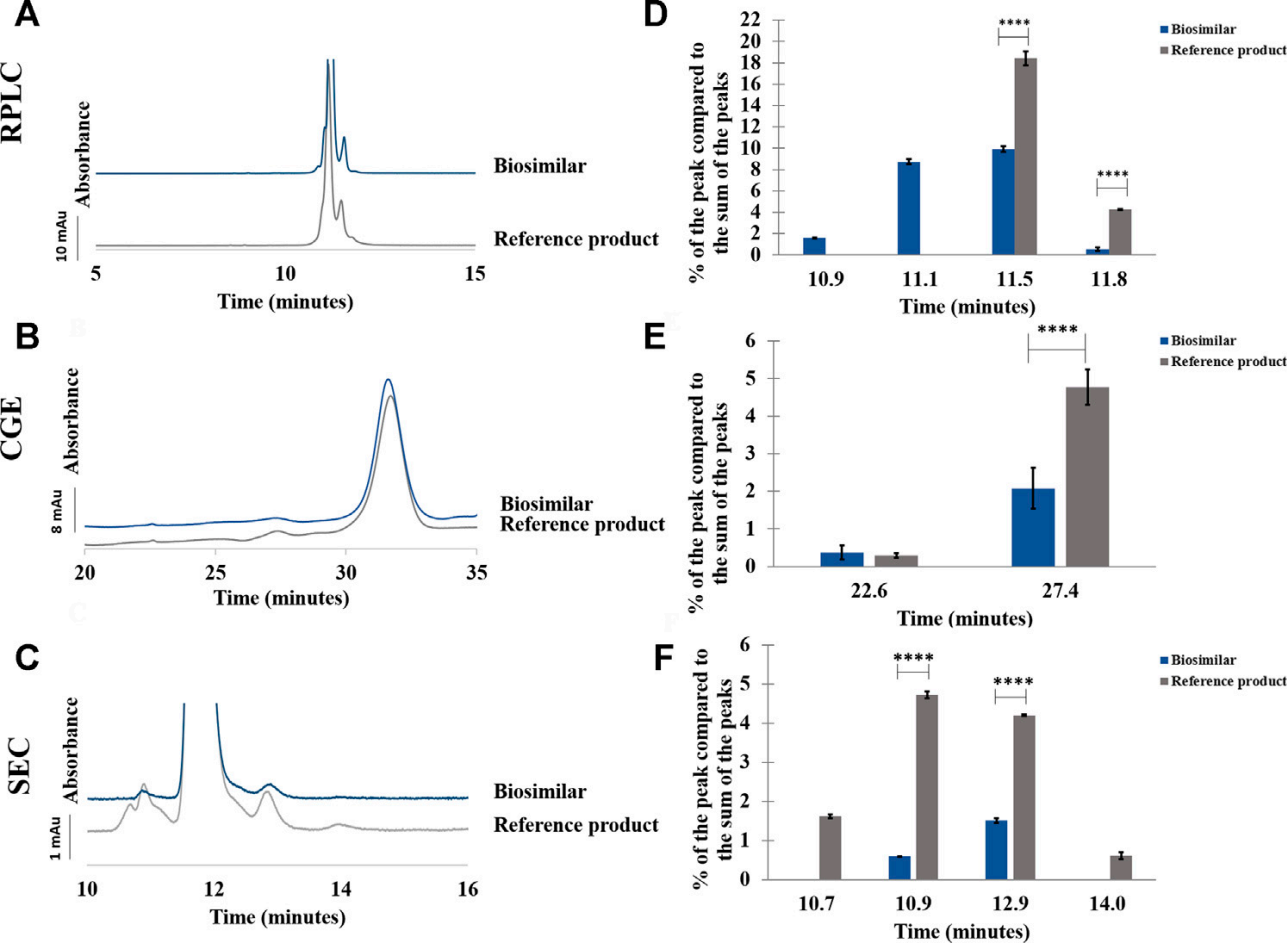
Comparison between etanercept reference product and its biosimilar by RPLC **(A–D)**, CGE **(B–E),** and SEC **(C–F)**. Chromatograms **(A–C)** and percentage of the peak compared to the sum of the peaks in function of the retention/migration time (n = 3) **(D–F)**. For CGE, the peak areas of the peaks were corrected by their migration times. **** represents statistically different results with a *p*-value <0.0001. See Material and methods for additional information on experimental conditions.

In RPLC, small differences were observed between the reference product and the biosimilar (see Figure 6A,D). A shoulder can be seen before the main peak in the biosimilar but not in the reference product. This shoulder corresponds to more hydrophilic species and represents 8.7% of the sum of the peak areas. Moreover, another hydrophilic species eluting just before the shoulder was also present only in the biosimilar and represented 1.6% of the sum of the peak areas. The percentage of the peak eluting just after the main peak (a 75-kDa fragment) was almost two times higher in the reference product than in the biosimilar (see Supplementary Table S3). RSDs below 0.1% were found for the retention time of the main peak and below 0.5% for its peak area for both the biosimilar and the reference product (see Supplementary Table S4).

Samples of etanercept were also analyzed by RPLC-QTOF to determine the exact mass of the main peak of the reference product and that of the biosimilar. Unfortunately, no exact mass measurement could be extracted from the acquired data. Indeed, the high heterogeneity of Fc-fusion proteins due to the presence of multiple glycosylation sites makes the analysis by MS particularly challenging (Duivelshof et al., 2021). The exact mass measurement of the protein is, thus, not possible without prior deglycosylation with an enzyme such as PNGase (Zhu et al., 2014).

In CGE, a higher percentage of the lower MW species was observed in the reference product compared to the biosimilar (4.8 vs. 2.1%) (see Figure 6B,E; Supplementary Table S3). The RSDs were below 0.2% for the migration time of the main peak and below 3.7% for its peak area (see Supplementary Table S4).

In SEC, a lower quantity of fragments and aggregates was found in the biosimilar compared with the reference product. Moreover, two groups of fragments and aggregates were observed for the reference product although there was only one group of fragments and aggregates for the biosimilar (see Figures 6C,F). A higher proportion of fragments (6.4% vs. 0.6%) and higher percentage of aggregates (4.8% vs. 1.5%) were observed in the reference product compared with the biosimilar (see Supplementary Table S3). RSDs below 0.1% were found for the retention time of the main peak and below 0.4% for its peak area (see Supplementary Table S4).

As in the case of adalimumab, no further degradation was observed for both samples subjected to a temperature of 30°C and a stirring at 300 rpm for 4 h (data not shown).

## 4 Conclusion and Perspectives

With the increasing number of antibodies and derivatives on the market and the emergence of biosimilars, there is an important need for methods to study the comparability between the reference products and their biosimilars. The three analytical techniques (RPLC, SEC, and CGE) used herein to study adalimumab, etanercept, and their biosimilars were found to be very complementary.

The forced degradation applied to the studied biopharmaceuticals produced fragments and aggregates. It was shown that, although SEC is interesting for the detection of aggregates, CGE is able to distinguish between the fragments of adalimumab. However, it failed to give information about fragments for etanercept. RPLC provided information about the relative hydrophobicity of degradation products. RPLC and SEC were more repeatable than CGE. However, RSDs of retention/migration times were always lower than 0.5%.

The comparison between the biosimilars and their reference products showed that they are highly similar, but some slight differences were observed. A greater quantity of fragments and aggregates was observed in the adalimumab biosimilar compared with the reference product, but a smaller quantity of aggregates and fragments was observed in the etanercept biosimilar compared with the reference product. Similar results were obtained for the samples subjected to mild stress conditions.

In this work, complementary techniques to study size-variant products (SEC and CGE) and hydrophilic and hydrophobic impurities (RPLC) were used. Other chromatographic and electrophoretic techniques could also be used to evaluate comparability. Ion-exchange chromatography and capillary isoelectric focusing would be useful to study acidic and basic variants, and HILIC and CZE would help determine the glycosylation profile of the biopharmaceuticals (Berkowitz et al., 2012; Fekete et al., 2015; Pedersen-Bjergaard et al., 2019). All those variants need to be carefully controlled as they represent critical quality attributes that may affect product safety and efficacy (Oshinbolu et al., 2018).

Mass spectrometry was useful to determine the exact mass of the intact protein of adalimumab and could also be employed to determine the mass of the impurities. Due to the complexity of the glycosylation profile of etanercept, the use of an enzyme to cleave glycosidic bonds would be necessary to simplify the spectrum and enable analysis of the intact protein (Zhu et al., 2014).

Analysis by SEC and CGE showed a difference between the retention/migration times of these two biopharmaceuticals. The MW of etanercept was overestimated using both techniques knowing that the estimation of MW relies for both techniques on a calibration curve, including an IgG antibody standard. This difference is most probably due to the high glycosylation of etanercept which renders MW estimation inaccurate.

The combination of SEC with MALS detection was used to measure the MW of etanercept (150 kDa) without overestimation. MALS has the advantage of determining the MW of proteins without the need for reference molecules having the same conformation. It is considered an absolute technique that determines the MW of compounds according to the amount of light scattered by them (Some et al., 2019).

Ion mobility spectrometry would also be an interesting additional technique to provide information on the three-dimensional conformation of adalimumab and etanercept. Moreover, collision-induced unfolding analysis could be applied to study how the molecules unfold when submitted to an accelerating electric field (Kerr et al., 2019).

Besides the analytical comparison of reference products with their related biosimilars, a biological activity comparison between these products must also be performed to demonstrate that they have similar pharmacological activity.

The analysis of monoclonal antibodies has been described in many publications due to the high number of mAbs on the market and the increasing number of biosimilars, but fewer articles dealing with Fc-fusion proteins can be found. Fc-fusion proteins are more complex molecules than mAbs as their structure depends on the structure of the ligand that is bound to the Fc fragment. As this ligand is different for every commercialized Fc-fusion protein, it is probably not possible to develop generic methods as is the case for mAbs. Moreover, Fc-fusion proteins present often more complex glycosylation profiles than those observed for mAbs, and innovative analytical methods are, therefore, required (Duivelshof et al., 2021).

## Data Availability

The raw data supporting the conclusion of this article will be made available by the authors without undue reservation.
