# Supplementary material for: Comparison of Three Complementary Analytical Techniques for the Evaluation of the Biosimilar Comparability of a Monoclonal Antibody and an Fc-Fusion Protein
Source: Front Chem. 2021 Dec 6;9:782099. doi: 10.3389/fchem.2021.782099 (PMC8686473; doi:10.3389/fchem.2021.782099)
Supplement: Supplementary file 1 [file DataSheet1.docx]

Supplementary Material

## Supplementary Figures

**Supplementary Figure 1.** CGE calibration curve made with proteins of 20, 35 and 148 kDa .

##
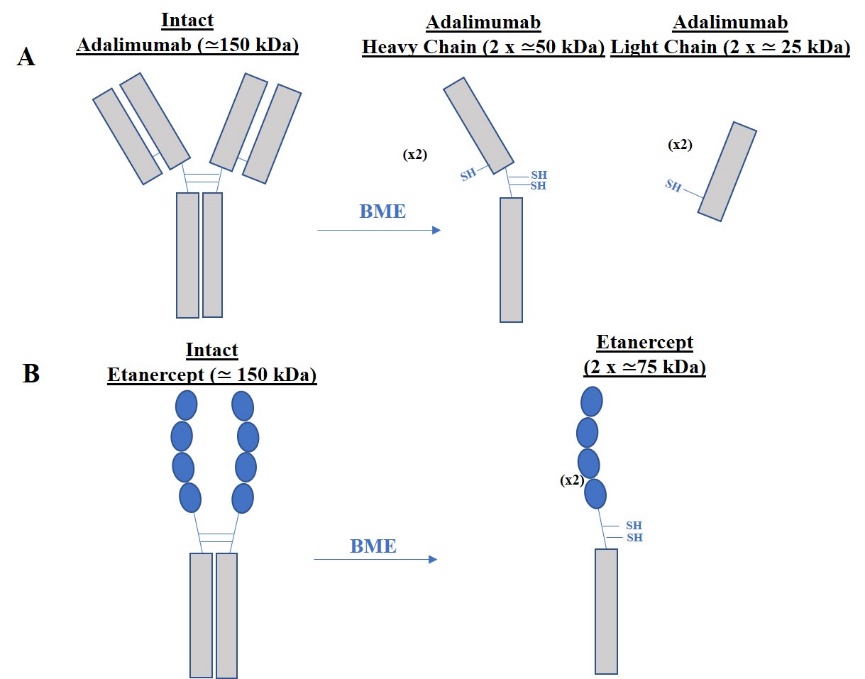


## Supplementary Figure 2. Schematic representation of adalimumab (A) and etanercept (B) reduction with BME.

##
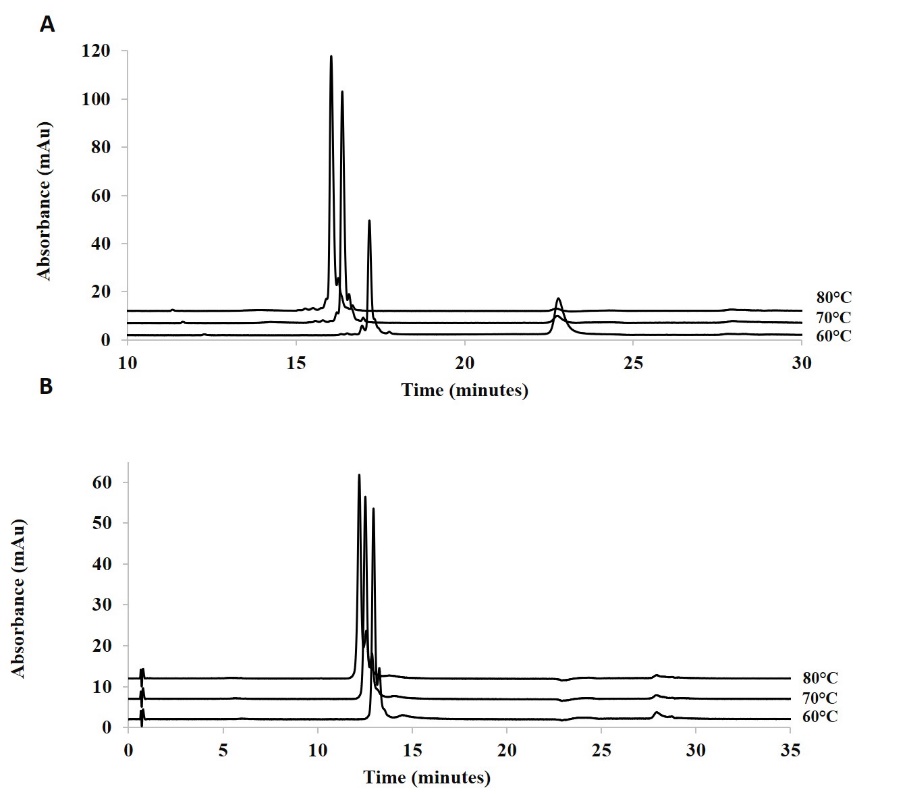


**Supplementary Figure 3.** Influence of the temperature on adsorption of adalimumab (A) and etanercept (B) in RPLC. Comparison of 60 °C, 70 °C and 80 °C column temperature on BioResolve (polyphenyl) column. See Material and methods for additional information on chromatographic conditions.

|  | **Adalimumab** | | | |
| --- | --- | --- | --- | --- |
|  | **Reference product** | | **Biosimilar** | |
| **RPLC** | **Retention time (min)** | **% of the peak compared to the sum of the peak areas ± SD (n=3)** | **Retention time (min)** | **% of the peak compared to the sum of the peak areas ± SD (n=3)** |
|  | 8.8 | 0.01 ± 0.00 | 8.7 | 0.02 ± 0.00 |
|  | 9.2 | 0.02 ± 0.00 | 9.1 | 0.05 ± 0.00 |
|  | 9.8 | 0.02 ± 0.00 | 9.7 | 0.01 ± 0.00 |
|  | 10.0 | 0.04 ± 0.00 | 9.9 | 0.03 ± 0.00 |
|  | 10.1 | 0.07 ± 0.00 | 10.0 | 0.28 ± 0.01 |
|  | 10.2 | 0.09 ± 0.01 | 10.1 | 0.06 ± 0.00 |
|  | 12.0 | 2.59 ± 0.21 | 11.9 | 1.08 ± 0.22 |
|  | 13.6 | 0.61 ± 0.03 | 13.5 | 0.65 ± 0.02 |
|  | 13.8 | 1.02 ± 0.01 | 13.7 | 1.09 ± 0.03 |
|  | 14.5 (main peak) | / | 14.3 (main peak) | / |
|  | 14.8 | 11.77 ± 0.29 | 14.6 | 14.09 ± 0.26 |
|  | 15.2 | 1.35 ± 0.10 | 15.0 | 1.53 ± 0.08 |
| **CGE** | **Migration time (min)** | **% of the peak compared to the sum of the peak areas ± SD (n=3)** | **Migration time (min)** | **% of the peak compared to the sum of the peak areas ± SD (n=3)** |
|  | 15.7 | 1.40 ± 0.31 | 16.1 | 1.16 ± 0.12 |
|  | 24.9 | 1.43 ± 0.20 | 25.5 | 1.09 ± 0.09 |
|  | 26.5 | 4.21 ± 0.63 | 27.1 | 5.73 ± 0.41 |
|  | 27.6 (main peak) | / | 28.4 (main peak) | / |
|  | / | / | 29.1 | 8.59 ± 0.37 |
| **SEC** | **Retention time (min)** | **% of the peak compared to the sum of the peak areas ± SD (n=3)** | **Retention time (min)** | **% of the peak compared to the sum of the peak areas ± SD (n=3)** |
|  | / | / | 11.0 | 0.42 ± 0.02 |
|  | 12.0 | 0.47 ± 0.02 | 11.7 | 2.22 ± 0.02 |
|  | 13.8 (main peak) | / | 13.8 (main peak) | / |
|  | 14.8 | 0.79 ± 0.04 | 14.8 | 0.87 ± 0.02 |
|  | 17.3 | 0.27 ± 0.06 | 17.2 | 0.26 ± 0.01 |

## Supplementary Tables

**Supplementary Table 1**. Migration and retention times of the peaks detected in the reference product and the biosimilar of adalimumab by RPLC, CGE and SEC, provided with their respective abundance in % compared to the sum of the peak areas (n=3). For CGE, the peak areas of the peaks were corrected by their migration times.

**Supplementary Table 2**. Comparison of the relative standard deviations for retention / migration times and peak areas of the main peak in the reference product and the biosimilar of adalimumab using the three analytical techniques (RPLC, CGE and SEC) (n=3).

*: RSD calculated from the ratio of the peak area of the main peak to its migration time and corrected with the ratio of the peak area of the internal standard (lysozyme) to its migration time.

|  | **Adalimumab** | | | | |
| --- | --- | --- | --- | --- | --- |
|  | **Reference product** | | **Biosimilar** | | |
|  | **RSD for retention / migration time (min)** | **RSD for peak area (mAu)** | **RSD for retention / migration time (min)** | **RSD for peak area (mAu)** |  |
| **RPLC** | 0.25% | 0.21% | 0.13% | 0.10% |  |
| **CGE** | 0.45% | 9.14%* | 0.01% | 4.32%* |  |
| **SEC** | 0.02% | 0.13% | 0.03% | 0.03% |  |

**Supplementary Table 3.** Migration and retention times of the peaks detected in the reference product and the biosimilar of etanercept by RPLC, CGE and SEC, provided with their respective abundance in % compared to the sum of the peak areas (n=3). For CGE, the peak areas of the peaks were corrected by their migration times.

|  | **Etanercept** | | | | |
| --- | --- | --- | --- | --- | --- |
|  | **Reference product** | | **Biosimilar** | |  |
| **RPLC** | **Retention time (min)** | **% of the peak compared to the sum of the peak areas ± SD (n=3)** | **Retention time (min)** | **% of the peak compared to the sum of the peak areas ± SD (n=3)** |  |
|  | / | / | 10.9 | 1.58 ± 0.05 |  |
|  | / | / | 11.1 | 8.73 ± 0.22 |  |
|  | 11.1 (main peak) | / | 11.2 (main peak) | / |  |
|  | 11.5 | 18.42 ± 0.66 | 11.6 | 9.91 ± 0.27 |  |
|  | 11.7 | 4.27 ± 0.06 | 11.8 | 0.55 ± 0.14 |  |
| **CGE** | **Migration time (min)** | **% of the peak compared to the sum of the peak areas ± SD (n=3)** | **Migration time (min)** | **% of the peak compared to the sum of the peak areas ± SD (n=3)** |  |
|  | 22.7 | 0.30 ± 0.06 | 22.6 | 0.37 ± 0.19 |  |
|  | 27.5 | 4.77 ± 0.47 | 27.4 | 2.08 ± 0.54 |  |
|  | 31.8 (main peak) | / | 31.6 (main peak) | / |  |
| **SEC** | **Retention time (min)** | **% of the peak compared to the sum of the peak areas ± SD (n=3)** | **Retention time (min)** | **% of the peak compared to the sum of the peak areas ± SD (n=3)** |  |
|  | 10.7 | 1.62 ± 0.05 | / | / |  |
|  | 10.9 | 4.73 ± 0.09 | 10.9 | 0.60 ± 0.01 |  |
|  | 11.7 (main peak) | / | 11.7 (main peak) | / |  |
|  | 12.8 | 4.20 ± 0.02 | 12.9 | 1.52 ± 0.05 |  |
|  | 14.0 | 0.62 ± 0.09 | / | / |  |

**Supplementary Table 4.** Comparison of the relative standard deviations for retention / migration times and peak areas of the main peak in the reference product and the biosimilar of etanercept using the three analytical techniques (RPLC, CGE and SEC) (n=3).

*: RSD calculated from the ratio of the peak area of the main peak to its migration time and corrected with the ratio of the peak area of the internal standard (lysozyme) to its migration time.

|  | **Etanercept** | | | |
| --- | --- | --- | --- | --- |
|  | **Reference product** | | **Biosimilar** | |
|  | **RSD on retention / migration time (min)** | **RSD on peak area (mAu)** | **RSD on retention / migration time (min)** | **RSD on peak area (mAu)** |
| **RPLC** | 0.07% | 0.52% | 0.11% | 0.29% |
| **CGE** | 0.23% | 2.40%* | 0.14% | 3.69%* |
| **SEC** | 0.03% | 0.36% | >0.01% | 0.15% |
